# Evaluation of Chelator-to-Antibody Ratio on Development of ^89^Zr-iPET Tracer for Imaging of PD-L1 Expression on Tumor

**DOI:** 10.3390/ijms242417132

**Published:** 2023-12-05

**Authors:** Shih-Chuan Tsai, Shiou-Shiow Farn, Wei-Lin Lo, Fang-Yu Ou Yang, Yong-Ching Kang, Liang-Cheng Chen, Kuo-Ting Chen, Jiunn-Wang Liao, Jui-Yin Kung, Jenn-Tzong Chen, Feng-Yun J. Huang

**Affiliations:** 1 Department of Nuclear Medicine, Taichung Veterans General Hospital, Taichung 407219, Taiwan; sctsai@vghtc.gov.tw (S.-C.T.); jykung@vghtc.gov.tw (J.-Y.K.); 2 National Atomic Research Institute, Taoyuan 325207, Taiwan; amanda@nari.org.tw (S.-S.F.); loweilin@nari.org.tw (W.-L.L.); wy6687@gmail.com (F.-Y.O.Y.); lcchen@nari.org.tw (L.-C.C.); jezon@nari.org.tw (J.-T.C.); 3 Department of Medical Imaging and Radiological Sciences, Central Taiwan University of Science and Technology, Taichung 406053, Taiwan; kangs0147@gmail.com; 4 Department of Chemistry, National Dong Hwa University, Hualien 974301, Taiwan; ktchen26@gms.ndhu.edu.tw; 5 Graduate Institute of Veterinary Pathobiology, National Chung-Hsing University, Taichung 402202, Taiwan; jwliao@dragon.nchu.edu.tw

**Keywords:** ^89^Zr, DFO, iPET, PD-L1/PD-1, chelator-to-antibody ratio

## Abstract

^89^Zr-iPET has been widely used for preclinical and clinical immunotherapy studies to predict patient stratification or evaluate therapeutic efficacy. In this study, we prepared and evaluated ^89^Zr-DFO-anti-PD-L1-mAb tracers with varying chelator-to-antibody ratios (CARs), including ^89^Zr-DFO-anti-PD-L1-mAb_3X (**tracer_3X**), ^89^Zr-DFO-anti-PD-L1-mAb_10X (**tracer_10X**), and ^89^Zr-DFO-anti-PD-L1-mAb_20X (**tracer_20X**). The DFO-anti-PD-L1-mAb conjugates with varying CARs were prepared using a random conjugation method and then subjected to quality control. The conjugates were radiolabeled with ^89^Zr and evaluated in a PD-L1-expressing CT26 tumor-bearing mouse model. Next, iPET imaging, biodistribution, pharmacokinetics, and ex vivo pathological and immunohistochemical examinations were conducted. LC–MS analysis revealed that DFO-anti-PD-L1-mAb conjugates were prepared with CARs ranging from 0.4 to 2.0. Radiochemical purity for all tracer groups was >99% after purification. The specific activity levels of **tracer_3X**, **tracer_10X**, and **tracer_20X** were 2.2 ± 0.6, 8.2 ± 0.6, and 10.5 ± 1.6 μCi/μg, respectively. ^89^Zr-iPET imaging showed evident tumor uptake in all tracer groups and reached the maximum uptake value at 24 h postinjection (p.i.). Biodistribution data at 168 h p.i. revealed that the tumor-to-liver, tumor-to-muscle, and tumor-to-blood uptake ratios for **tracer_3X**, **tracer_10X**, and **tracer_20X** were 0.46 ± 0.14, 0.58 ± 0.33, and 1.54 ± 0.51; 4.7 ± 1.3, 7.1 ± 3.9, and 14.7 ± 1.1; and 13.1 ± 5.8, 19.4 ± 13.8, and 41.3 ± 10.6, respectively. Significant differences were observed between **tracer_3X** and **tracer_20X** in the aforementioned uptake ratios at 168 h p.i. The mean residence time and elimination half-life for **tracer_3X**, **tracer_10X**, and **tracer_20X** were 25.4 ± 4.9, 24.2 ± 6.1, and 25.8 ± 3.3 h and 11.8 ± 0.5, 11.1 ± 0.7, and 11.7 ± 0.6 h, respectively. No statistical differences were found between-tracer in the aforementioned pharmacokinetic parameters. In conclusion, ^89^Zr-DFO-anti-PD-L1-mAb tracers with a CAR of 1.4–2.0 may be better at imaging PD-L1 expression in tumors than are traditional low-CAR ^89^Zr-iPET tracers.

## 1. Introduction

The recently emerged immune checkpoint blockade therapies have revolutionized cancer treatment. Their high therapeutic efficacy and manageable side effects make them a promising option for cancer treatment [1]. Immune checkpoint molecules are primarily associated with various physiological functions, such as regulating the balance between the immune system and autoimmunity in humans [2,3]. In addition, these checkpoint molecules play key roles between immune and tumor cells. They can inhibit the activation of immune cells, such as T cells, B cells, and macrophages, by binding to the corresponding immune checkpoint molecules, making the immune system unable to identify tumor cells and thus increasing the tumor survival rate [4,5,6,7,8,9]. Currently, the most commonly investigated immune checkpoint molecules include cytotoxic T-lymphocyte-associated antigen 4 (CTLA-4), programmed cell death ligand 1 (PD-L1; also known as B7-H1), and programmed cell death protein 1 (PD-1). Notably, the PD-L1/PD-1 axis has been most interesting to researchers in the past decade. PD-1 is a 55-kDa transmembrane protein and is mainly expressed in cells, including activated T-cells, B lymphocytes, natural killer (NK) cells, macrophages, monocytes, dendritic cells, and myeloid-derived suppressor cell (MDSCs). By contrast, PD-L1 belongs to the B7 family of ligands and is a 33-kDa transmembrane glycoprotein in its extracellular region as the ligand of PD-1. Activated T-cells, macrophages, B-cells, dendritic cells, some epithelial cells, and tumor cells usually express PD-L1. The binding of PD-L1/PD-1 between tumor cells and T-cells can induce adaptive immune mechanisms to escape anti-tumor responses [10]. On the basis of the aforementioned mechanisms, researchers have successfully developed immune checkpoint inhibitors (ICI) for cancer treatment. An ICI monoclonal antibody (mAb) can block the interaction between checkpoint molecules on immune and tumor cells, thereby reactivating the patients’ immune system to target and kill tumor cells [4,8]. Since the anti-CTLA-4 ICI (ipilimumab) were first approved by the US Food and Drug Administration in 2011, nine ICI mAbs, including three anti-PD-L1 (durvalumab, atezolizumab, and avelumab), and six anti-PD-1 (cemiplimab, sintilimab, toripalimab, pembrolizumab, nivolumab, and camrelizumab) agents have been approved as standard treatments for >24 different types of cancer and tissue-agnostic indications [11,12,13,14,15,16,17,18,19,20,21]. As of 2022, more than 5683 active clinical trials (with 82% of them testing combination regimens) involving PD-L1/PD-1 ICI mAbs have been conducted worldwide [21]. Furthermore, research is ongoing to investigate novel immune checkpoint and costimulatory molecules, such as VISTA, LAG-3, TIM-3, and IDO1 [22,23].

Although ICI have been widely used for cancer therapy in clinical practice, the low response rate is a predominant challenge that needs to be addressed. In the United States, although 43.6% of all patients with cancer are eligible for ICI therapy, only 12.5% of patients respond to it [24]. Studies have reported that the expression level of PD-L1 in tumors is highly correlated with patients’ response to ICI therapies. Therefore, the detection of PD-L1 expression in tumors before or after treatment may be beneficial for patient stratification and treatment efficacy evaluation [25,26,27]. Currently, immunohistochemistry (IHC) remains the gold standard for measuring the expression level of PD-L1 in tumor tissues [28]. However, tumor-biopsy-based IHC assays have several limitations, including the static and heterogeneous expression of PD-L1 in tissues, invasiveness of the sampling procedure, and possibility of sampling errors. Moreover, PD-L1 expression is dynamic and can change over time in response to anticancer treatments, such as ICI therapies, chemotherapy, and radiotherapy [29,30,31,32,33,34,35]. Thus, novel imaging probes are needed for precisely detecting PD-L1 expression in tumors.

The success of ICI mAbs in cancer treatment has widened the application of immuno-positron emission tomography (iPET) in the field of nuclear medicine. iPET combines the high binding specificity of antibodies with the high sensitivity of positron emission tomography, enabling a noninvasive, whole-body, and dynamic evaluation of PD-L1 expression in tumors. This quantitative and molecular imaging approach can be valuable for assessing patient stratification with ICI therapies and obtain mechanistic insights into cancer biology [35,36,37,38]. ^89^Zr-iPET plays a crucial role in immunotherapy because of its innate characteristics, such as a physical half-life of 78.41 h and a β^+^ decay of 23%, which make it a suitable tracer for the imaging of antibodies and the precise evaluation of drug pharmacokinetics and biodistribution in vivo [35,39,40,41,42]. Recently, several PD-L1/PD-1 ICI mAb–based ^89^Zr-iPET tracers have been investigated in clinical trials [43,44,45,46,47,48,49] and reviewed in relevant studies [41,50].

The initial step in the preparation of ^89^Zr-iPET tracers involves the development of an immunoconjugate. This bioconjugation process can be approximately categorized into random (lysine-based) and site-specific (cysteine- and enzyme-based) methods. From a chemistry viewpoint, a lysine-based bioconjugation method is more accessible than a site-specific method because of the presence of approximately 90 lysine residues in a typical IgG1 human antibody, with most of them being chemically accessible. Moreover, the high reproducibility and efficiency of the lysine-based method make it an attractive choice for the clinical production of immunoconjugates [51]. Thus, the random method is currently the most common approach for preparing immunoconjugates, which are used to produce ^89^Zr-iPET tracers for use in preclinical and clinical investigations. However, a notable drawback of the random conjugation method is that it yields heterogeneous immunoconjugates in contrast to the site-specific method, which produces homogeneous immunoconjugates [52]. An essential parameter for antibody–drug conjugates is the drug-to-antibody ratio or chelator-to-antibody ratio (CAR), which quantifies the number of payloads or chelators attached to an antibody molecule. The drug-to-antibody ratio/CAR can affect the stability, immunoreactivity, pharmacokinetics, and biodistribution of immunoconjugates in vivo [52]. In previous preclinical and clinical studies, a CAR of approximately 0.3–1.0 was most commonly used to prepare ^89^Zr-iPET tracers [43,53,54,55]. However, to the best of our knowledge, no study has determined the optimal CAR for enhancing the efficacy of the antibody-based ^89^Zr-iPET tracer.

**Scheme 1:**
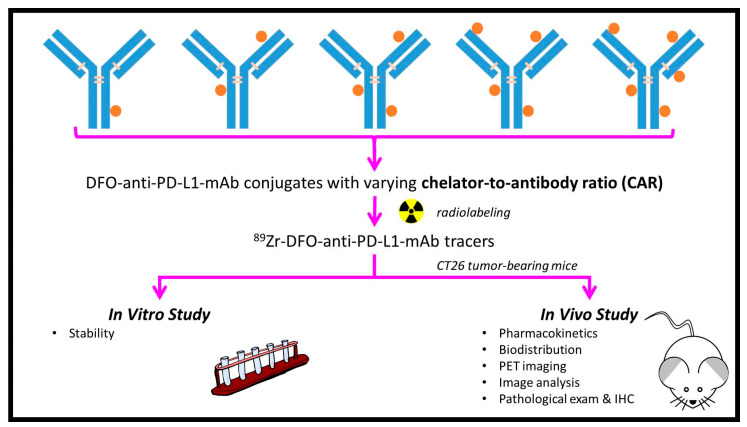
Flowchart depicting the steps of our study.

In this study, we prepared and characterized ^89^Zr-p-SCN-Bn-deferoxamine (DFO)-anti-PD-L1-mAb tracers with varying CARs. These tracers were then examined using a mouse model bearing CT26 tumors that expressed PD-L1 (Scheme 1). We performed in vivo studies, including iPET imaging and biodistribution and pharmacokinetic analyses, as well as ex vivo studies, including pathological and immunohistochemistry examinations.

## 2. Results

### 2.1. Preparation and Characterization of DFO-Anti-PD-L1-mAb Conjugates

**Figure 1:**
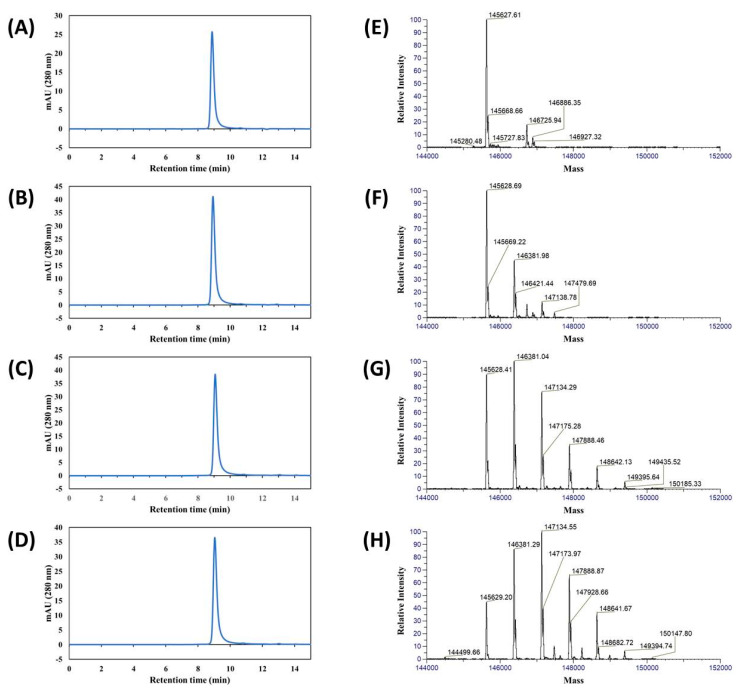
SE-HPLC chromatograms and LC–MS spectra of the prepared DFO-anti-PD-L1-mAb conjugates. The SE-HPLC chromatograms are presented as follows: (**A**) unmodified standard anti-PD-L1-mAb, (**B**) DFO-anti-PD-L1-mAb conjugate_3X, (**C**) DFO-anti-PD-L1-mAb conjugate_10X, and (**D**) DFO-anti-PD-L1-mAb conjugate_20X. The LC–MS spectra are presented as follows: (**E**) unmodified standard anti-PD-L1-mAb, (**F**) DFO-anti-PD-L1-mAb conjugate_3X, (**G**) DFO-anti-PD-L1-mAb conjugate_10X, and (**H**) DFO-anti-PD-L1-mAb conjugate_20X.

DFO-anti-PD-L1-mAb conjugates with varying numbers of chelators were prepared using the traditional conjugation method. Subsequently, we conducted quality control assessments and determined the CARs of the conjugates through size exclusion-high-performance liquid chromatography (SE-HPLC) and liquid chromatography–mass spectrometry (LC–MS). Table 1 presents the preparation conditions and quality control results for the prepared DFO-anti-PD-L1-mAb conjugates. The average CARs of DFO-anti-PD-L1-mAb conjugate_3X, DFO-anti-PD-L1-mAb conjugate_10X, and DFO-anti-PD-L1-mAb conjugate_20X were 0.4 (Figure 1F), 1.4 (Figure 1G), and 2.0 (Figure 1H), respectively. All conjugates were stored in 0.2 M HEPES buffer (pH of 7.0–7.5) at 4 °C for subsequent radiolabeling studies. Figure 1 presents the chromatograms and mass spectra of the conjugates, as obtained through SE-HPLC and LC–MS, respectively. The SE-HPLC spectra revealed that all conjugates were transparent, with no aggregation and 100% chemical purity (i.e., monomer content; Figure 1B–D). The mass spectra revealed a heterogeneous pattern of the conjugates, with CARs varying from 0 to 6.

### 2.2. Radiolabeling and In Vitro Stability Study

**Figure 2:**
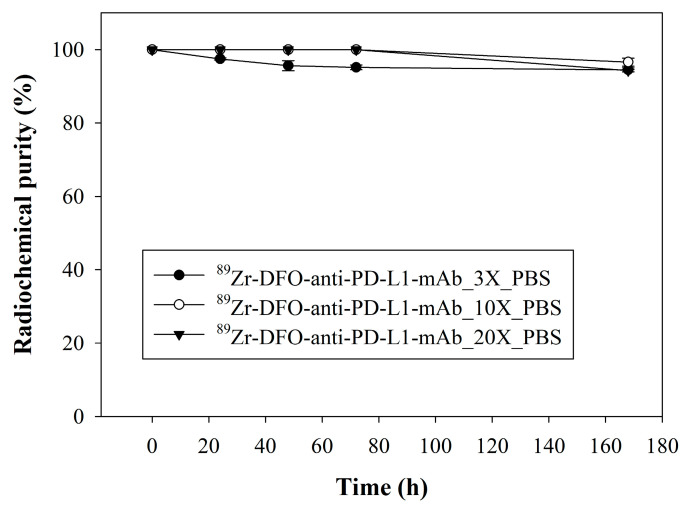
In vitro stability of the ^89^Zr-DFO-anti-PD-L1-mAb tracers incubated in PBS (mean ± SD, *n* = 3). (●) **tracer_3X**, (○) **tracer_10X**, and (▼) **tracer_20X**.

Table 2 presents the specifications for various ^89^Zr-DFO-anti-PD-L1-mAb tracers. Upon visual inspection, we noted that the final product solutions for all tracers were transparent, clear, and without aggregation. Instant thin layer chromatography/silica gel (ITLC/SG) revealed that the radiochemical yields of **tracer_3X**, **tracer_10X**, and **tracer_20X** were 24.3% ± 7.1%, 91.1% ± 3.2%, and 98.7% ± 0.5%, respectively. Furthermore, the radiochemical purity of all tracer groups was >99% after purification (Figure S1). The radio-SE-HPLC confirmed the radiochemical identity and purity of the tracers (Figure S2). The amounts of radioimpurities and aggregation at the end of synthesis were <2% for all tracers. The specific activity levels of **tracer_3X**, **tracer_10X**, and **tracer_20X** were 2.2 ± 0.6, 8.2 ± 0.6, and 10.5 ± 1.6 μCi/μg, respectively. In the in vitro stability study, ITLC/SG revealed that the radiochemical purity levels of all tracers decreased only slightly after 7 days of incubation in phosphate-buffered saline (PBS). The radiochemical purity levels of **tracer_3X**, **tracer_10X**, and **tracer_20X** remained at 94.5% ± 0.6%, 96.6% ± 1.1%, and 94.3% ± 0.3%, respectively (Figure 2).

### 2.3. ^89^Zr-iPET Imaging

**Figure 3:**
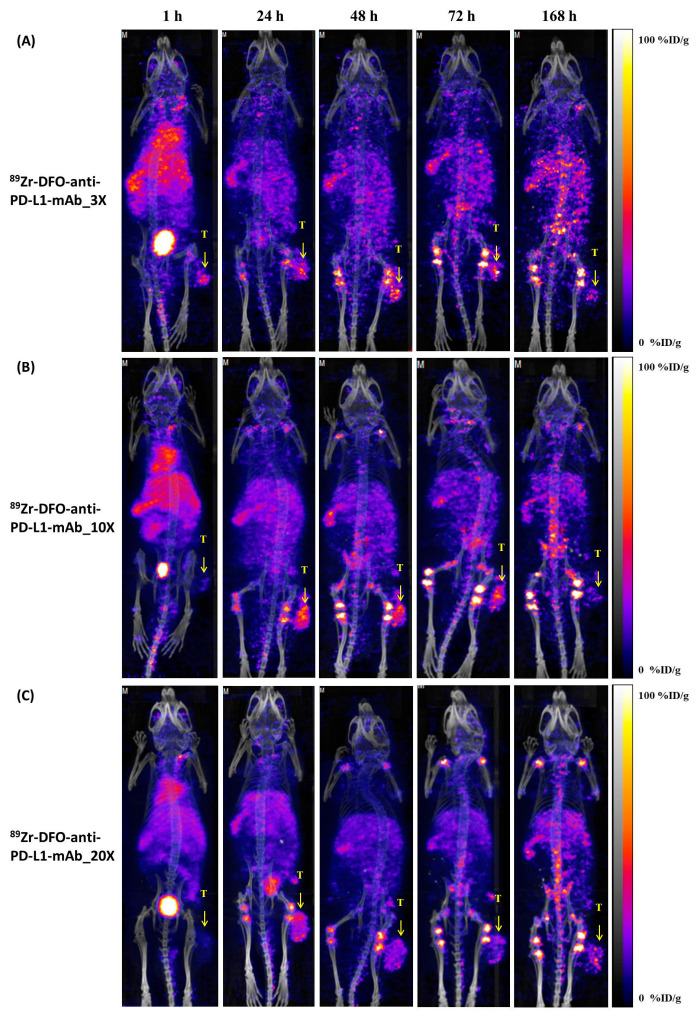
^89^Zr-iPET imaging of PD-L1 expression in CT26 tumor-bearing mice injected with various ^89^Zr-DFO-anti-PD-L1-mAb tracers. The images were obtained at 1, 24, 48, 72, and 168 h p.i. (intravenous); (**A**) **tracer_3X**, (**B**) **tracer_10X**, and (**C**) **tracer_20X**. (yellow arrows and letter ‘T’ mean tumor).

**Figure 4:**
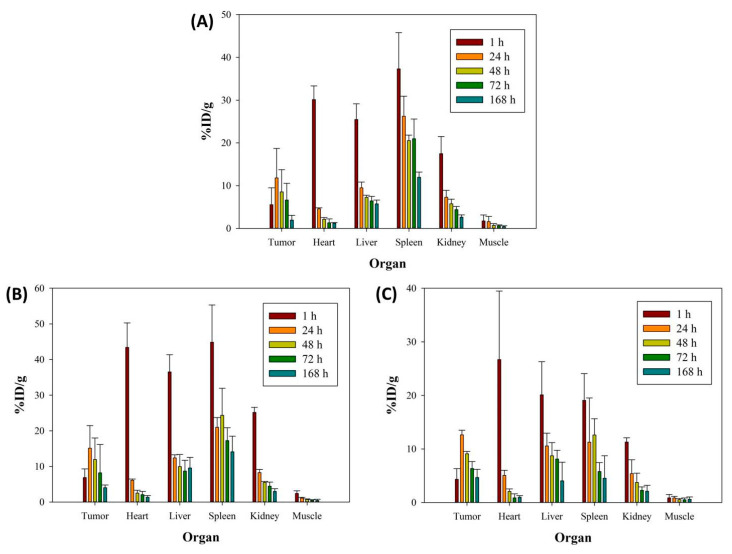
Biodistribution of ^89^Zr-DFO-anti-PD-L1-mAb tracers in a PD-L1-expressing CT26 tumor-bearing mouse model at 1, 24, 48, 72, and 168 h p.i. The results of an ROI analysis of ^89^Zr-iPET images (mean ± SD, *n* = 3); (**A**) **tracer_3X**, (**B**) **tracer_10X**, and (**C**) **tracer_20X**.

Figure 3 presents the iPET scans of CT26 tumor-bearing mice injected with various ^89^Zr-DFO-anti-PD-L1-mAb tracers (**tracer_3X**, **tracer_10X**, and **tracer_20X**). The results of iPET imaging clearly revealed tumor uptake for all tracers, with the most distinct tumor images observed at approximately 24 to 48 h post-injection (p.i.; Figure 3A–C). In addition to tumors, some normal organs such as the liver, spleen, and bone absorbed the tracers. Notably, tracer uptake in the bladder was evident at 1 h p.i. The region of interest (ROI) analysis of iPET images indicated that the highest tumor uptake at 24 h p.i. for **tracer_3X**, **tracer_10X**, and **tracer_20X** was 11.8 ± 6.91, 15.11 ± 6.35, and 12.63 ± 0.86 percentage of injection dose per gram of tissues (%ID/g), respectively. The ROI analysis further revealed that tracer uptake in nontarget organs declined gradually (Figure 4). The tumor-to-muscle (T/M) uptake ratio reached the maximum value at 48 h p.i.: 12.21-, 16.37-, and 18.35-fold for **tracer_3X**, **tracer_10X**, and **tracer_20X**, respectively. Subsequently, the T/M uptake ratio decreased after 48 h p.i. until the end point. Thus, **tracer_20X** exhibited the strongest tumor imaging ability, representing an approximately 1.5-fold improvement in tumor imaging contrast compared with that noted with **tracer_3X**.

### 2.4. Biodistribution and Pharmacokinetics

**Figure 5:**
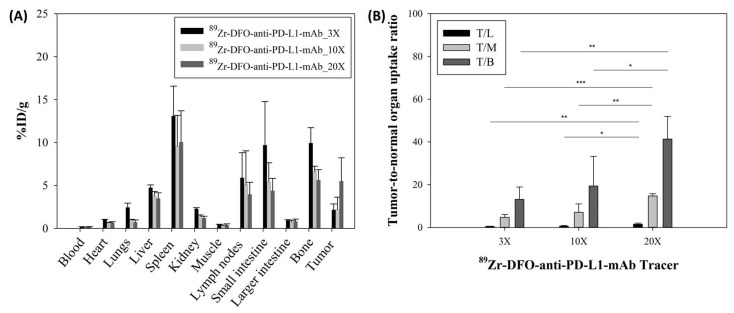
Biodistribution of the ^89^Zr-DFO-anti-PD-L1-mAb tracers in PD-L1-expressing CT26 tumor-bearing mice at 168 h p.i. (mean ± SD, *n* = 4). (**A**) Percentage of injection dose per gram of organ or tissue (%ID/g) in various tissues/organs for different ^89^Zr-DFO-anti-PD-L1-mAb tracers. (**B**) Tumor-to-normal organ uptake ratios (T/L: tumor-to-liver; T/M: tumor-to-muscle; and T/B: tumor-to-blood) for different ^89^Zr-DFO-anti-PD-L1-mAb tracers (3X, 10X, and 20X). * *p* < 0.05, ** *p* < 0.01, and *** *p* < 0.001.

**Figure 6:**
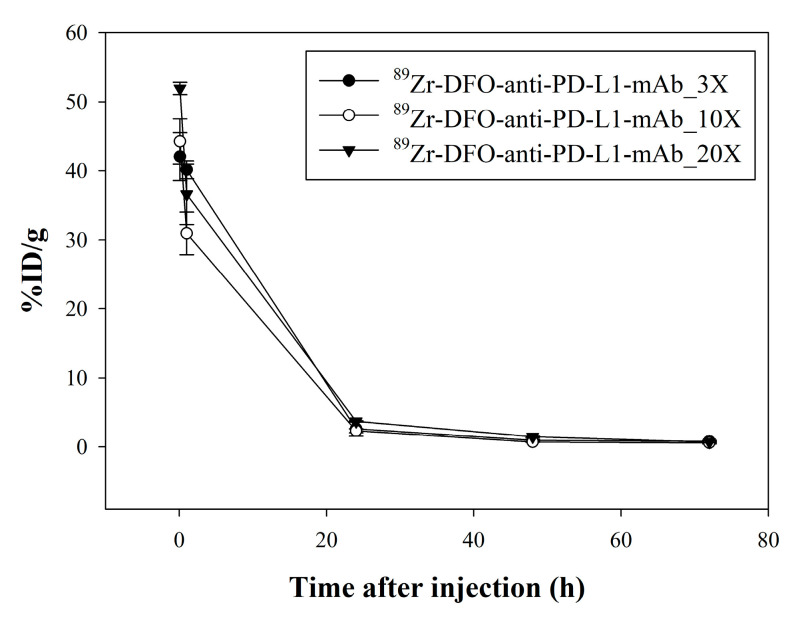
Clearance curves of the ^89^Zr-DFO-anti-PD-L1-mAb tracers from the blood of CT26 tumor-bearing mice (mean ± SD, *n* = 4). (●) **tracer_3X**; (○) **tracer_10X**; and (▼) **tracer_20X**.

Figure 5A illustrates the biodistribution results of various ^89^Zr-DFO-anti-PD-L1-mAb tracers in PD-L1-expressing CT26 tumor-bearing mice at 168 h p.i. The tumor uptake values of **tracer_3X**, **tracer_10X**, and **tracer_20X** were 2.2 ± 0.7, 2.2 ± 1.4, and 5.5 ± 2.7%ID/g at 168 h p.i., respectively (Table S1). The maximum accumulation of the tracers was observed in the spleen, with values of 13.1 ± 3.5, 9.6 ± 3.6, and 10.0 ± 3.7%ID/g for **tracer_3X**, **tracer_10X**, and **tracer_20X**, respectively. In addition, prominent tracer uptake was observed in the lymph nodes and bones, with uptake values of 5.9 ± 2.9, 5.1 ± 3.9, and 4.0 ± 1.4%ID/g and 9.9 ± 1.8, 6.6 ± 0.6, and 5.6 ± 1.2%ID/g for **tracer_3X**, **tracer_10X**, and **tracer_20X**, respectively (Table S1). Figure 5B presents the tumor-to-normal organ (T/N) uptake ratios, including the tumor-to-liver (T/L), T/M, and tumor-to-blood (T/B) ratios, derived from the ex vivo biodistribution data. All T/N (T/L, TM, and T/B) uptake ratios increased with increasing CARs (from 0.4 to 2.0). Thus, the T/L, T/M, and T/B uptake ratios for **tracer_3X**, **tracer_10X**, and **tracer_20X** were 0.46 ± 0.14, 0.58 ± 0.33, and 1.54 ± 0.51; 4.7 ± 1.3, 7.1 ± 3.9, and 14.7 ± 1.1; and 13.1 ± 5.8, 19.4 ± 13.8, and 41.3 ± 10.6, respectively. Significant between-group differences were noted in the T/L uptake ratio—**tracer_20X** versus **tracer_10X** (*p* = 0.0189) and **tracer_20X** versus **tracer_3X** (*p* = 0.0063), T/M uptake ratio—**tracer_20X** versus **tracer_10X** (*p* = 0.0094) and **tracer_20X** versus **tracer_3X** (*p* = 0.00002), and the T/B uptake ratio—**tracer_20X** versus **tracer_10X** (*p* = 0.0455) and **tracer_20X** versus **tracer_3X** (*p* = 0.0035). Figure 6 displays the clearance curves of the ^89^Zr-DFO-anti-PD-L1-mAb tracers from the blood of CT26 tumor-bearing mice. Table 3 presents the estimated pharmacokinetic parameters of the clearance curves of the ^89^Zr-DFO-anti-PD-L1-mAb tracers. The mean residence time (MRT) and elimination half-life (T_1/2_) for **tracer_3X**, **tracer_10X**, and **tracer_20X** were 25.4 ± 4.9, 24.2 ± 6.1, and 25.8 ± 3.3 h and 11.8 ± 0.5, 11.1 ± 0.7, and 11.7 ± 0.6 h, respectively. No significant between-group differences (*p* > 0.05) were noted in the MRT or T_1/2_; the *p* values for the correlation between **tracer_20X** and **tracer_10X**, between **tracer_20X** and **tracer_3X**, and between **tracer_10X** and **tracer_3X** were 0.7019, 0.9098, and 0.7969 and 0.3591, 0.7512, and 0.2284, respectively.

### 2.5. Pathological Examination and IHC

**Figure 7:**
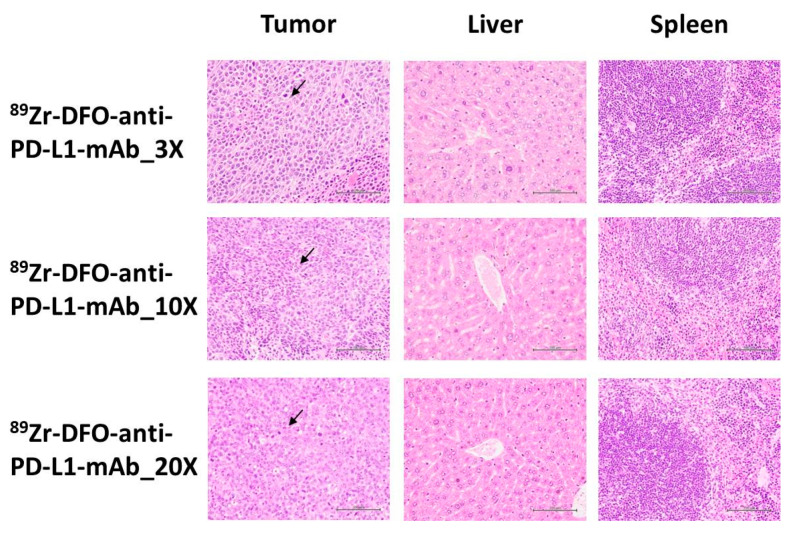
Histopathology of CT26 tumor-bearing mice at 7 days p.i. On tumoral histopathological slides (400×), survived tumor cells were detected as epithelioid cells with high mitotic activity (arrow) and multifocal necrosis in the tracer groups, respectively. The black bar indicates the scale bar: 100 μm. No significant changes were noted in the liver or spleen. Hematoxylin and eosin staining.

**Figure 8:**
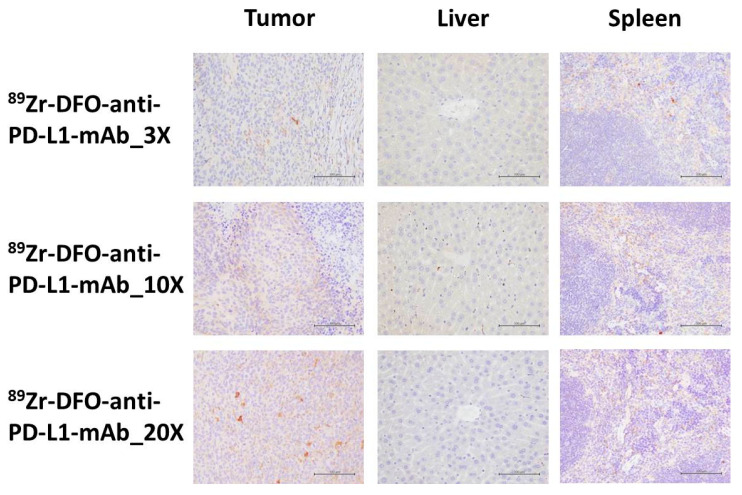
Immunohistochemical expression of PD-L1 in CT26 tumor-bearing mice at 7 days p.i. On tumoral and splenic immunohistochemical slides (400×), PD-L1 expression was detected on the basis of DAB reactions around the cells. The black bar indicates the scale bar: 100 μm. No significant PD-L1 expression was noted in the liver. IHC staining.

Figure 7 presents the results of the histopathological examinations of the tumor, liver, and spleen of CT26 tumor-bearing mice; the examinations were performed 7 days p.i. Tumor cells exhibited a dense, circular-to-oval-shaped morphology with high mitotic activity; the adjacent normal tissues were compressed. However, no significant changes were noted in the liver or spleen. Figure 8 presents the results of an IHC assay performed to evaluate the expression of PD-L1 in CT26 tumor-bearing mice at 7 days p.i. On the tumor tissue slide, PD-L1 staining was visualized as a brown 3,3′-Diaminobenzidine (DAB) signal, revealing the heterogeneous expression of PD-L1. In addition, a similar DAB signal was observed on the spleen tissue slide. However, no noticeable DAB reaction was observed on the liver tissue slide.

## 3. Discussion

In the process of preparing DFO-anti-PD-L1-mAb conjugates, we observed a substantial decline in protein recovery and precipitated protein level when the molar excess of the DFO-to-antibody ratio exceeded 40-folds during conjugation. This finding indicates that the overloading of hydrophobic DFO onto the antibody reduces the solubility of the final product, leading to antibody aggregation in centrifuge tubes and a low protein recovery. The LC–MS analysis confirmed that the prepared conjugates had varying CARs, indicative of a heterogeneous pattern achieved through the random conjugation method. Furthermore, the average CAR ranged from 0.4 to 2.0 when the molar excess of the DFO-to-antibody ratio was 3–20-folds in the conjugation reaction. To prepare ^89^Zr-iPET tracers, studies have commonly used the formulations of DFO-anti-PD-L1-mAb conjugates with an average CAR ranging from 0.3 to 1.0 [43,53,54,55]. Some studies have used higher average CARs, ranging from 1 to 4 [56,57,58,59]. For the random conjugation method, the lowest possible average CAR should be considered to minimize its effects on antibody immunoreactivity and stability; however, it is also needed high enough to provide satisfied imaging ability. The site-specific method was recently used to prepare ^89^Zr-iPET tracers with a homogeneous CAR of 2 or 4. This technique resulted in superior tumor imaging ability compared with that of the traditional low-CAR method. Moreover, tracers with a CAR of 2 appear to outperform those with a CAR of 4 [60,61].

In this study, we used an excess amount of ^89^Zr activity for radiolabeling DFO-anti-PD-L1-mAb conjugates with ^89^Zr to prepare tracers with the maximum specific activity. The radiochemical yields were 24.3% ± 7.1%, 91.1% ± 3.2%, and 98.7% ± 0.5% for DFO-conjugate_3X (CAR of 0.4), DFO-conjugate_10X (CAR of 1.4), and DFO-conjugate_20X (CAR of 2.0), respectively. The radiochemical yield of the tracers increased significantly with a gradual increase in CAR, and the radiochemical purity of each tracer group was >99% after purification. Specific activity was the highest (10.5 ± 1.6 μCi/μg) in the **tracer_20X** group (CAR of 2.0). However, the ^89^Zr source used in this study was purchased from overseas and required approximately 2 days for transportation. Therefore, the theoretical specific activity of **tracer_20X** may reach 16.15 μCi/μg when considering the decay correction of ^89^Zr. Based on the decay correction mentioned above, it is similar to an optimized (on the basis of modified 1–3 DFO per mAb) ^89^Zr-immuno-PET tracer with a specific activity of 17.5 ± 2.2 μCi/μg, as described in a previous study [59].

iPET scans revealed that each tracer successfully depicted the tumor image at all imaging time points. The clearest tumor image was observed at approximately 24–48 h p.i. (Figure 3), consistent with the findings of previous studies [62,63]. In addition to tumor uptake, tracers accumulated in normal organs, including the liver, spleen, and small intestine. These nontarget accumulation of tracers may be associated with the expression of PD-L1 in normal organs [62,64] or the elimination of mAbs from the body [65]. The accumulation of the tracer in the bone is related to the instability of the ^89^Zr-DFO complex in vivo, resulting in the dissociation of free ^89^Zr^4+^ ions that seek the bone tissue [39,66]. Notably, the biodistribution results obtained from the semiquantitative ROI analysis of the images revealed that the uptake of tracers by tumor tissues initially increased and then decreased after reaching the maximum uptake at 24 h p.i. (Figure 4). This pattern of tracer uptake suggests that specific binding occurred between the tracers and PD-L1-expressing CT26 tumor cells. By contrast, tracer uptake in other normal organs/tissues gradually decreased (i.e., nonspecific binding). Furthermore, the T/M uptake ratios of **tracer_3X** and **tracer_20X** at 24 h and 48 h p.i. were 7.4 and 16.5 and 12.2 and 18.4, respectively. This finding indicates that the image contrast between T/M_(**tracer_3X**)_ and T/M_(**tracer_20X**)_ at 24 h and 48 h was increased by 2.22- and 1.50-folds, respectively. These findings suggest that **tracer_20X**, with its relatively high specific activity (10.5 ± 1.6 μCi/μg), can enhance the tumor imaging ability compared with that of the traditional **tracer_3X** with a relatively low CAR (2.2 ± 0.6 μCi/μg). Many studies have reported that blocking experiments involving the coinjection or preinjection of unlabeled anti-PD-L1-mAb in excess (3–100-folds) with the tracer protein during ^89^Zr-iPET scans may enhance tumor imaging characteristics and reduce tracer uptake in PD-L1-expressing nontarget organs and tissues, such as the spleen and lymph nodes [49,56,62,63,67,68]. However, even in the absence of blocking experiments during ^89^Zr-iPET imaging in this study, clear tumor images were observed in different tracer groups. In summary, the development of ^89^Zr-DFO-anti-PD-L1-mAb tracers with an optimal CAR, such as a CAR of 1.4 to 2.0, for imaging PD-L1 expression in tumors may improve the specific activity of the tracers, tumor accumulation of the tracers, and contrast of tumor imaging.

The biodistribution data collected at 168 h p.i. revealed that tracer uptake by tumor tissues decreased to <6%ID/g. However, the tumor uptake of **tracer_20X** was 2.53-fold and 2.48-fold higher than that of **tracer_3X** (*p* = 0.053) and **tracer_10X** (*p* = 0.076), respectively. The tumor imaging ability of **tracer_20X** demonstrated that its T/N uptake ratio was higher than that of **tracer_10X** and **tracer_3X**, with significant differences observed in T/L, TM, and T/B between the tracers (Figure 5B). These findings are also similar to those of the iPET scan. Moreover, the pharmacokinetic analysis revealed that **tracer_20X** with a CAR of 2.0 significantly maintained the MRT and T_1/2_ at values similar to those of **tracer_10X** or **tracer_3X** (Table 3). This finding suggests that **tracer_20X** with a CAR of 2.0 can retain good long-circulation characteristics in vivo, similar to stable traditional low-CAR tracers. Therefore, **tracer_20X** with a CAR of 2.0, which results in high specific activity, may have stronger tumor imaging ability than traditional low-CAR tracers do. In addition to tumor uptake, some normal organs such as the spleen, lymph nodes, and small intestine absorbed the tracers; these findings are consistent with the results of ^89^Zr-iPET imaging experiments. The nontarget uptake can be attributed to PD-L1 expression in some normal organs and the elimination of mAbs in vivo. Notably, the results of biodistribution in this study are similar to those of other studies examining the uptake of ^89^Zr-DFO-anti-PD-L1-mAb tracers in nontarget organs rich in PD-L1, such as the heart, spleen, lymph nodes, intestines, pancreas, and skin [67,68]. However, the effects of tracer accumulation in normal organs on the imaging of PD-L1 expression in tumors remain unclear and require further investigation. To explain the specific binding between the ^89^Zr-DFO-anti-PD-L1-mAb probe and the PD-L1 ligand on CT26 tumor cells, we performed an IHC assay for tumors and normal organs at the end of the animal study. On the tumor tissue slide, PD-L1 expression was clearly evident as a brown DAB signal, demonstrating the heterogeneous expression of PD-L1 (Figure 8). This suggests that the accumulation of ^89^Zr-DFO-anti-PD-L1-mAb tracers in CT26 tumors is correlated with specific binding. By contrast, low PD-L1 expression in normal organs, such as the liver, did not exhibit obvious DAB precipitation.

The present study demonstrates the effects of various CARs on the development of ^89^Zr-iPET tracers for the imaging of PD-L1 expression in tumors. ^89^Zr-DFO-anti-PD-L1-mAb tracers with a CAR of 2.0 were found to have a stronger tumor imaging ability than traditional low-CAR tracers do. However, bone uptake due to the in vivo instability of the radiolabeled ^89^Zr-DFO-complex may lead to false-positive results in bone metastasis. Recently, a site-specific radiolabeled technique based on glycan modification (CAR of 2 or 4) to prepare ^89^Zr-iPET tracers has emerged and demonstrated superior immunoreactivity, in vivo stability, and tumor uptake compared with traditional randomly radiolabeled low-CAR ^89^Zr-iPET tracers [60,61]. In light of the aforementioned findings, we recommend that the following factors be considered during the development of the next-generation optimal ^89^Zr-iPET tracers to enhance imaging performance: selection of the CAR, method of bioconjugation, choice of the bifunctional chelator, and coinjection of cold-mAb.

## 4. Materials and Methods

### 4.1. Materials

DFO was purchased from Macrocyclics (Plano, TX, USA). Amicon Ultra 0.5-mL 50-kDa centrifugal filters for DNA and protein purification and concentration measurement were purchased from Merck (Darmstadt, Germany). Zeba Spin Desalting columns (40 K MWCO and 0.5-mL volume) were obtained from Thermo Fisher (Cambridge, MA, USA). Chelex 100 Resin was purchased from Bio-Rad (Hercules, CA, USA), and diethylenetriaminepentaacetic acid was obtained from Sigma-Aldrich (St. Louis, MO, USA). InVivoMAb anti-mouse PD-L1 was purchased from Bioxcell (Lebanon, NH, USA). Gibco fetal bovin serum, penicillin/streptomycin, Dulbecco’s PBS (without Ca^2+^ and Mg^2+^), 0.25% Trypsin/EDTA solution, and Dulbecco’s Modified Eagle Medium were purchased from Thermo Fisher. [^89^Zr]-oxalate solution (21.73–37.13 mCi/mL) was obtained from PerkinElmer (Boston, MA, USA). Zenix-C SEC-300 (3 μm, 7.8 × 300-mm columns) and Zenix-C SEC-300 (3 μm, 7.8 × 50-mm columns) were purchased from Sepax Technologies (Newark, DE, USA). All others chemicals and reagents were obtained from Sigma-Aldrich.

### 4.2. Preparation and Characterization of DFO-Anti-PD-L1-mAb Conjugates

DFO-anti-PD-L1-mAb conjugates were prepared using the conventional conjugation method [53] with some modifications. In brief, DFO chelator solution was freshly prepared in dimethyl sulfoxide solution (7.5 mg/mL) and then added into 200–2000 μg of anti-PD-L1-mAb (5 mg/mL) in 0.1 M sodium carbonate solution (pH 9.0). Different molar excess DFO-to-antibody ratios were used: 3:1 (3X), 10:1 (10X), and 20:1 (20X). The mixed solution was reacted under shaking at 350 rpm at room temperature for 50 min. After the reaction was completed, the unreacted DFO chelator was removed through filtration by using Amicon Ultra 0.5-mL 50-kDa centrifugal filters. Then, the purified DFO-anti-PD-L1-mAb conjugate solution was subjected to quality control by using the Agilent 1100 SE-HPLC system (Agilent Technologies, Carpinteria, CA, USA). The CAR was analyzed using the Ultra3000 HPLC system and an orbitrap fusion Lumos mass spectrometry (ThermoFisher Scientific, San Jose, CA, USA).

### 4.3. Radiolabeling and In Vitro Stability Study

**Figure 9:**
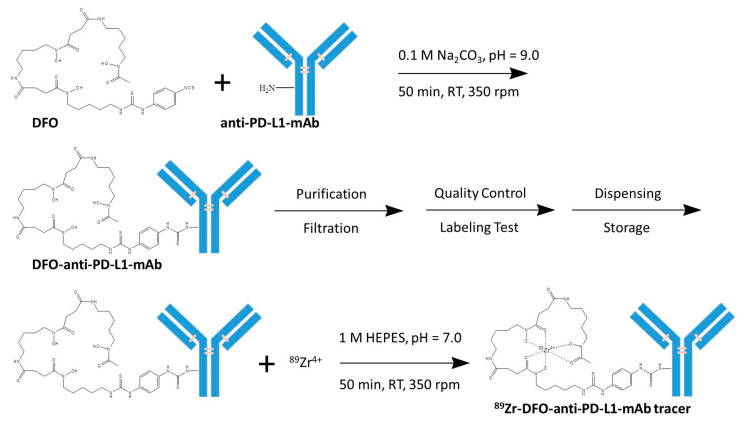
Radiolabeling of the ^89^Zr-DFO-anti-PD-L1-mAb tracers.

The DFO-anti-PD-L1-mAb conjugates with different CARs were radiolabeled with ^89^Zr, as reported previously (Figure 9) [53], with some modifications. First, an aliquot of 100–200 μg of DFO-anti-PD-L1-mAb conjugates (concentration: 5–10 μg/μL) was added into a 0.5-mL Eppendorf tube. Then, 37–148 MBq of ^89^Zr-oxalate solution was added into the conjugate solution. The mixture was reacted in the presence of 1M HEPES buffer (pH 7) under shaking condition (350 rpm) at room temperature for 50 min. An aliquot of 2–3 μL of 50 mM diethylenetriaminepentaacetic acid solution was added into each reaction tube to terminate the reaction; the reaction tubes were left undisturbed for another 5 min at room temperature. After the reaction was complete, the radiochemical yields of the ^89^Zr-DFO-anti-PD-L1-mAb tracers were analyzed using the ITLC/SG system. Subsequently, the unlabeled ^89^Zr-oxalate and possible impurities were removed by passing the mixture through spin desalting columns (40 K MWCO and 0.5 mL). The radiochemical purity levels of the tracers were analyzed through ITLC/SG and Radio-SE-HPLC (stationary phase: Zenix-C SEC-300 3 μm, 7.8 × 300 mm; mobile phase: 1× PBS, flow rate: 1 mL/min), respectively. In vitro stability of ^89^Zr-DFO-anti-PD-L1-mAb tracers with different CARs was measured in PBS under shaking condition (350 rpm) at room temperature. ITLC/SG was performed to analyze the radiochemical purity of each tracer in PBS at different time points, including 0, 24, 48, 72, and 168 h p.i.

### 4.4. Cell Culture and Animal Model Establishment

The murine colorectal cancer cell line CT26 was purchased from the American Type Culture Collection (Manassas, VA, USA) and cultured in Roswell Park Memorial Medium 1640 supplemented with 10% (*v*/*v*) fetal bovine serum, 2 mM l-glutamine, 100 units/mL penicillin, and 100 μM/mL streptomycin. The cells were incubated at 37 °C in a humidified environment with 5% CO_2_. To establish a CT26 tumor-bearing mouse model, normal BALB/c mice (4–6-week-old male mice) were obtained from BioLASCO Taiwan Co., Ltd. (Taipei, Taiwan). The mice were housed in a controlled environment and fed food and water ad libitum. CT26 murine colorectal carcinoma cells were harvested and subcutaneously injected (2 × 10^5^ cells/0.1 mL) the right hind leg of the mice. Tumor volume (mm^3^) was calculated using the formula = 0.5 × a × b^2^ (a = length; b = width). The tumor-bearing mice were used for experiments when the tumor volume reached approximately 150–200 mm^3^. All animal studies were approved by the Institutional Animal Care and Use Committee of the Institute of Nuclear Energy Research, Taoyuan, Taiwan (approval number: 111020).

### 4.5. Animal Positron Emission Tomography Imaging

^89^Zr-DFO-anti-PD-L1-mAb tracers with different CARs were used for the imaging of PD-L1 expression in CT26 tumor-bearing mice through nanoPET/CT (Bioscan Inc., Washington, DC, USA) [69,70]. Nine tumor-bearing mice were randomly divided into three groups (three per group) for the imaging study. For this, each group was intravenously injected with one type of tracer (1.2–5.6 MBq/0.1 mL fixed with protein at 8–10 μg): **tracer_3X**, **tracer_10X**, or **tracer_20X**. Before 5 min from nanoPET/CT imaging, the mice were anesthetized with 2% isoflurane (mixed with oxygen); during the PET/CT scan, anesthesia was maintained with 1% isoflurane. Then, the mice were placed in the prone position, and static PET imaging was performed for 60 min at 1, 24, 48, 72, and 168 h p.i. The list mode of image data processing was used for image reconstruction. For data analysis, the ROIs of the PET images were analyzed manually and the affected organ/tissue, including the heart, liver, spleen, kidney, muscle, lymph node, and tumor, was identified (*n* = 3). The average radioactivity concentration within a tumor or muscle region was calculated from the average pixel value within multiple ROI volumes. The ROIs were defined on co-registered PET/CT images by using the PMOD software (V3.4) to estimate radioactivity concentration. The counts in each ROI were converted to radioactivity per gram of tissue (nCi/g), assuming a tissue density of 1 g/mL, and then normalized to the %ID/g.

### 4.6. Biodistribution and Pharmacokinetics

Twelve CT26 tumor-bearing mice were used for the biodistribution and pharmacokinetics study. The mice were divided into three groups, with four mice in each group. Each group was intravenously injected with **tracer_3X**, **tracer_10X**, and **tracer_20X** (1.2–5.6 MBq/0.1 mL fixed with 8–10 μg protein, respectively). All mice in each group were euthanized at 168 h p.i.—the end point of PET imaging. The organs of interest, including the blood, heart, lungs, liver, spleen, kidney, muscle, lymph nodes, small intestine, large intestine, bone, and tumor, were dissected and weighed and then assessed for radioactivity by using PerkinElmer 2480 gamma counter (PerkinElmer, Waltham, MA, USA). Aliquots of the tracer injections were stored in advance as the standard initial injection doses. The results of the biodistribution assay are expressed in terms of %ID/g.

For the pharmacokinetics study, 10–20 μL of blood from each group was collected through tail vein puncture by using a 29-G needle. The radioactivity concentrations in the blood samples were measured using a gamma counter and expressed in terms of the percentage of injection dose per milliliter (%ID/mL). Pharmacokinetic parameters were determined using the WinNonlin software (version 8.3.4) (Pharsight: Mountain View, CA, USA). Noncompartmental analysis model 201 (IV-Bolus Input) and the log/linear trapezoidal rule were used. Pharmacokinetic parameters, including the maximum concentration, maximum concentration time, total body clearance, MRT, and T_1/2_, were determined.

### 4.7. Pathological and Immunohistochemistry Examinations

The mice were euthanized after the study end point. Subsequently, tissue and organs, including the tumor, liver, and spleen, were harvested and preserved in 10% formalin until subsequent pathological and IHC examinations. For the histopathological examination, hematoxylin and eosin staining was performed using the standard staining protocol. All IHC procedures (DS9800) were performed automatically using the Leica BOND-MAX Fully Automated IHC Staining System (Leica Biosystems, Deer Park, IL, USA). For this, 5-μm tissue sections were placed on suitable adhesive coating slides. The slides were heated at 50 °C for 15 min. The Bond Dewax solution was used (twice, 5 min each) to remove paraffin from the tissue sections and then washed (thrice, 5 min each) with Tris buffer saline (TBS; pH 7.6). The Bond Leica alcohol was used to rehydrate sections for 5 min and then washed (thrice, 5 min each) with TBS. Antigen retrieval was performed using Bond Epitope retrieval buffer, Bond EDTA buffer (pH 9.0), heated at 100 °C for 20 min and then washed (thrice, 5 min each) with TBS. The sections were treated with Bond Peroxide solution (3% hydrogen peroxide) for 8 min and then washed (thrice, 5 min each) with TBS. The sections were treated with Bond Protein Blocking solution for 10 min and then washed (thrice, 5 min each) with TBS. Primary antibodies were diluted (1:200; anti-PD-L1-mAb; ab233482) (Abcam, Waltham, MA, USA). The sections were incubated with primary antibodies for 30 min at room temperature and then washed (thrice, 5 min each) with TBS. Then, the sections were incubated in Bond Polymer solution (poly-HRP rabbit IgG) for 10 min and washed thrice in TBS for 5 min. Next, the sections were incubated (twice, 3 min each) with Bond DAB solution and then washed (thrice, 5 min each) with deionized water. The sections were counterstained with Bond Hematoxylin solution for 10 min and then washed (thrice, 5 min each) with deionized water. Finally, the sections were baked and mounted.

### 4.8. Statistical Analysis

All data are presented in terms of the mean ± standard deviation values. The unpaired Student *t* test was used for determining between-group differences. A *p* value of <0.05 was considered to be significant.

## 5. Conclusions

We successfully prepared and evaluated ^89^Zr-DFO-anti-PD-L1-mAb tracers with varying CARs. ^89^Zr-DFO-anti-PD-L1-mAb tracers with a CAR of 1.4 to 2.0 may have a stronger ability to image PD-L1 expression in colorectal tumors than traditional low-CAR ^89^Zr-iPET tracers. ^89^Zr-DFO-anti-PD-L1-mAb tracers with an optimal CAR (2.0) exhibit improved specific activity, tumor uptake, and tumor imaging contrast. Such tracers are expected to have in vivo stability and long-circulating characteristics similar to those of low-CAR ^89^Zr-iPET tracers. Our findings may facilitate the development of ^89^Zr-iPET tracers for clinical applications.

## Figures and Tables

**Table 1:** Preparation conditions and quality control results for DFO-anti-PD-L1-mAb conjugates.

DFO-Anti-PD-L1-mAb Conjugate	Reaction Condition				Quality Control			
	Temp. (°C)	pH	Reaction Time (min)	Molar Excess of DFO to Antibody (Fold)	Visual Inspection	pH	Chemical Yield (%)	Chelator-to-Antibody Ratio (Avg. CAR)
DFO-mAb conjugate_3X	25	9.0	50	3	transparent	7.0–7.5	100	0.4
DFO-mAb conjugate_10X	25	9.0	50	10	transparent	7.0–7.5	100	1.4
DFO-mAb conjugate_20X	25	9.0	50	20	transparent	7.0–7.5	100	2.0

**Table 2:** Specifications of the prepared ^89^Zr-DFO-anti-PD-L1-mAb tracers (mean ± SD, *n* = 3).

Subject	^89^Zr-DFO-Anti-PD-L1-mAb Tracers		
	Tracer_3X	Tracer_10X	Tracer_20X
Visual inspection	Transparent	Transparent	Transparent
Radiochemical yield (%)	24.3 ± 7.1	91.1 ± 3.2	98.7 ± 0.5
Radiochemical purity (%)	99.0 ± 0.8	100.0 ± 0.0	100.0 ± 0.0
Specific activity at the end of synthesis (μCi/μg)	2.2 ± 0.6	8.2 ± 0.6	10.5 ± 1.6
Protein dose injected for imaging study (μg)	8–10	8–10	8–10

**Table 3:** Pharmacokinetic parameters of the prepared ^89^Zr-DFO-anti-PD-L1-mAb tracers.

Parameter	Unit	Value of ^89^Zr-DFO-Anti-PD-L1-mAb Tracers		
		Tracer_3X	Tracer_10X	Tracer_20X
C_max_	%ID/g	42.0 ± 3.5	44.2 ± 3.3	51.9 ± 0.9
T_max_	h	0.083	0.083	0.083
Cl	g/h	0.22 ± 0.03	0.26 ± 0.02	0.20 ± 0.01
MRT	h	25.4 ± 4.9	24.2 ± 6.1	25.8 ± 3.3
T_1/2_	h	11.8 ± 0.5	11.1 ± 0.7	11.7 ± 0.6

C_max_, maximum concentration; T_max_, maximum concentration time; Cl, total body clearance; MRT, mean residence time; and T_1/2_, elimination half-life.

## Data Availability

Data are contained within the article and Supplementary Materials.

## Supplementary Materials

The supporting information can be downloaded at: https://www.mdpi.com/article/10.3390/ijms242417132/s1.
