# Supplementary material for: Evaluation of Chelator-to-Antibody Ratio on Development of 89Zr-iPET Tracer for Imaging of PD-L1 Expression on Tumor
Source: Int J Mol Sci. 2023 Dec 5;24(24):17132. doi: 10.3390/ijms242417132 (PMC10743313; doi:10.3390/ijms242417132)
Supplement: Supplementary file 1 [file ijms-24-17132-s001.zip › ijms-2709720-supplementary.pdf]

Supplementary data:

Table S1. Biodistribution of ^{89}Zr-DFO-anti-PD-L1-mAb tracers in PD-L1 expressed CT26 tumor-bearing mice model at168 h p.i. (mean ± SD, *n* = 4).

Organ/tissue	Uptake of ^{89}Zr-DFO-anti-PD-L1-mAb tracers (%ID/g)		
	**Tracer_3X**	**Tracer_10X**	**Tracer_20X**
Blood	0.17 ± 0.03	0.12 ± 0.03	0.14 ± 0.08
Heart	1.02 ± 0.05	0.66 ± 0.04	0.62 ± 0.17
Lungs	2.45 ± 0.50	1.00 ± 0.06	0.72 ± 0.26
Liver	4.73 ± 0.34	3.79 ± 0.51	3.48 ± 0.67
Spleen	13.08 ± 3.49	9.57 ± 3.60	10.03 ± 3.67
Kidney	2.29 ± 0.13	1.47 ± 0.11	1.20 ± 0.22
Muscle	0.46 ± 0.04	0.32 ± 0.08	0.37 ± 0.16
Lymph nodes	5.88 ± 2.93	5.10 ± 3.92	3.96 ± 1.41
Small intestine	9.69 ± 5.08	5.41 ± 2.22	4.37 ± 1.45
Large intestine	0.95 ± 0.07	0.74 ± 0.18	0.82 ± 0.27
Bone	9.93 ± 1.81	6.63 ± 0.60	5.62 ± 1.21
Tumor	2.17 ± 0.69	2.22 ± 1.42	5.50 ± 2.71

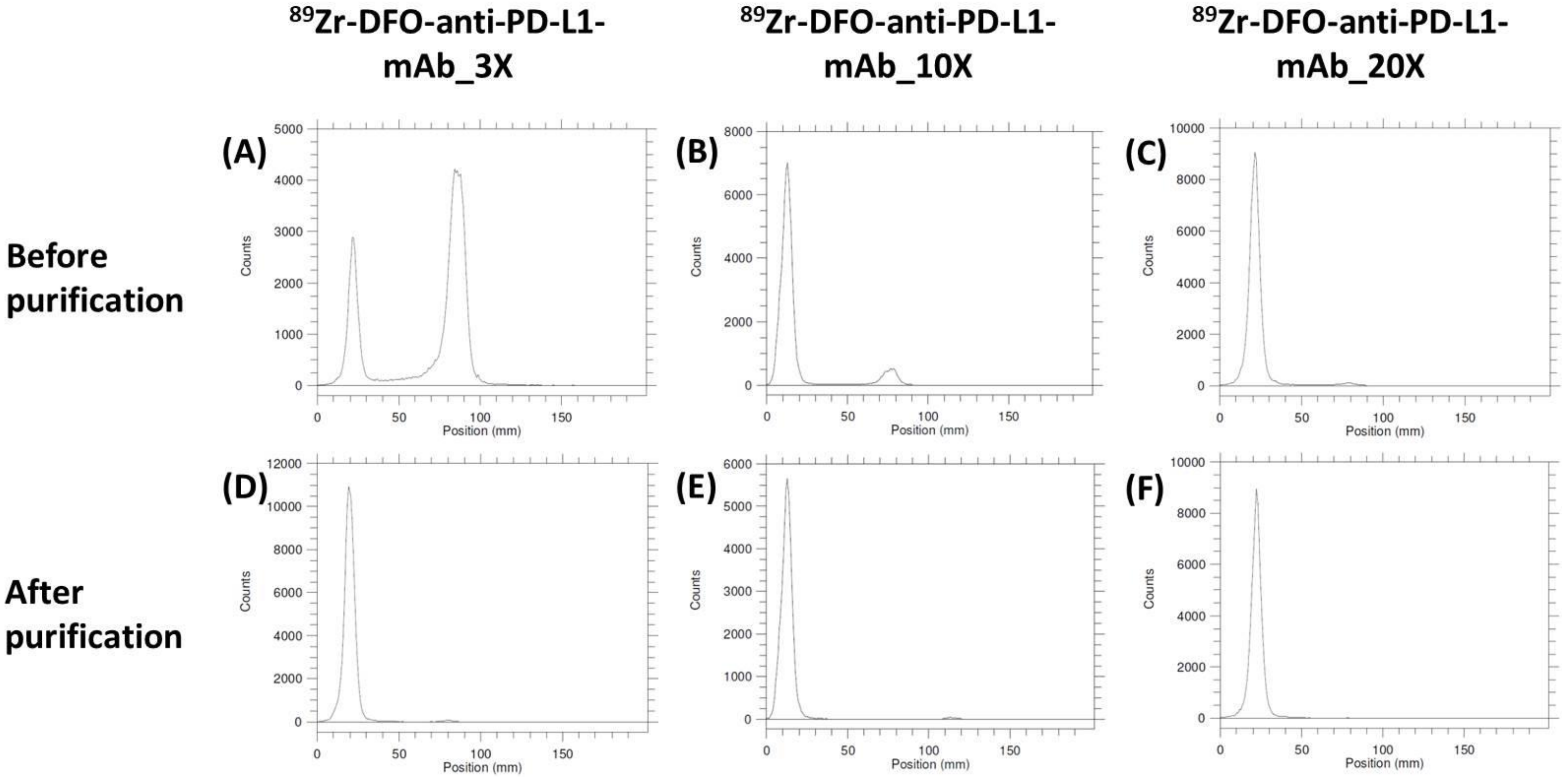

Figure S1. Radiochemical yield and purity analysis of ^{89}Zr-DFO-anti-PD-L1-mAb tracers by ITLC/SG. Final product solution before purification represented as (**A**) **tracer_3X**, (**B**) **tracer_10X**, and (**C**) **tracer_20X**. Final product solution after purification represented as (**D**) **tracer_3X**, (**E**) **tracer_10X**, and (**F**) **tracer_20X**.

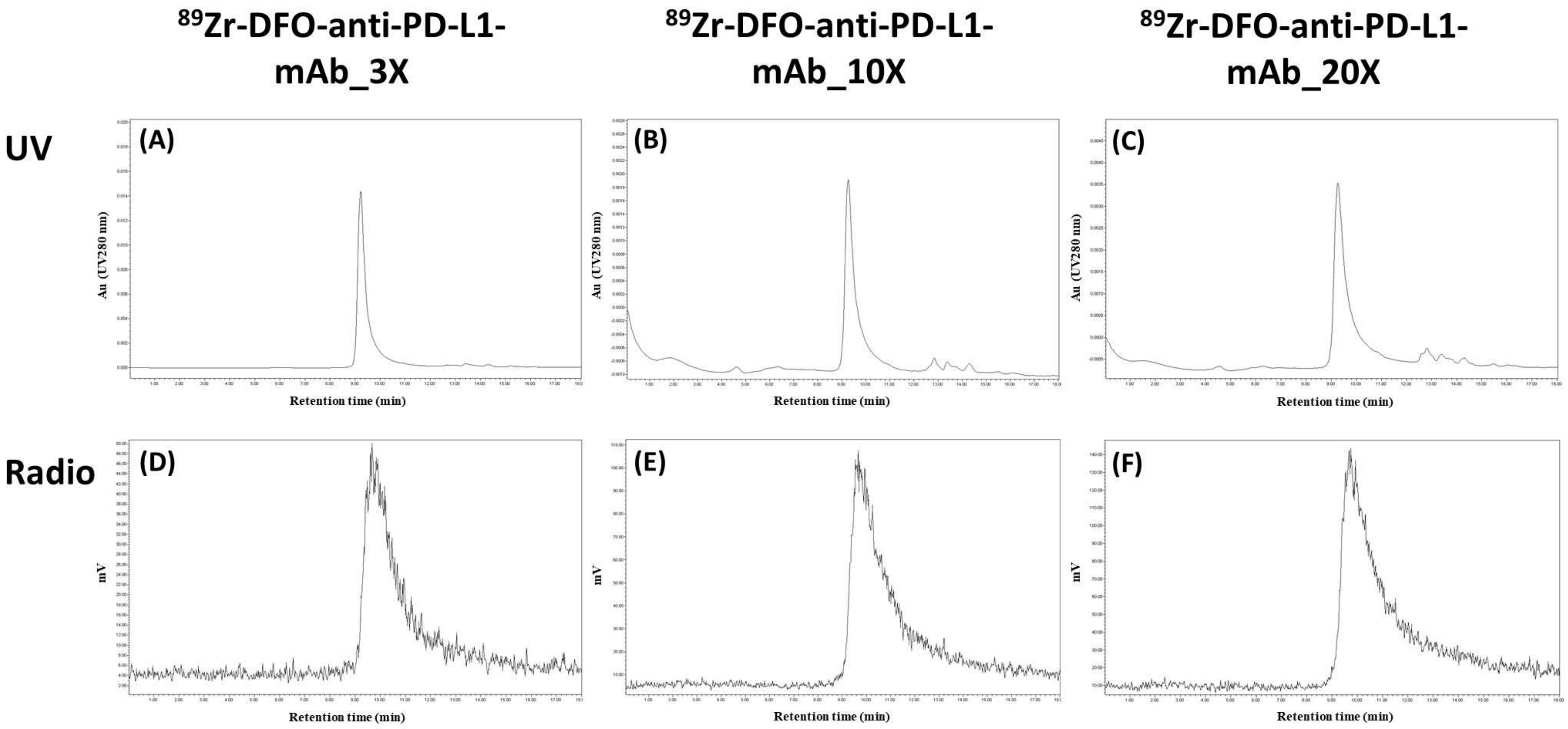

Figure S2. Radiochemical identity and purity analysis of ^{89}Zr-DFO-anti-PD-L1-mAb tracers by Radio-SE-HPLC. Signals from UV_{280nm} detector represented as (**A**) **tracer_3X**, (**B**) **tracer_10X**, and (**C**) **tracer_20X**. Signal from Radio-detector represented as (**D**) **tracer_3X**, (**E**) **tracer_10X**, and (**F**) **tracer_20X**.
